# ﻿*Paraphlomisjinggangshanensis* (Lamiaceae), a new species from Jiangxi, China

**DOI:** 10.3897/phytokeys.204.87654

**Published:** 2022-08-02

**Authors:** Wan-Yi Zhao, Zhong Zhang, Qiang Fan, Chun-Quan Chen, Wen-Bo Liao, David E. Boufford

**Affiliations:** 1 State Key Laboratory of Biocontrol and Guangdong Provincial Key Laboratory of Plant Resources, School of Life Sciences, Sun Yan-Sen University, Guangzhou 510275, China Sun Yan-Sen University Guangzhou China; 2 Changguling Forestry Farm, Jinggangshan Nature Reserve, Jinggangshan 343600, Jiangxi China Jinggangshan Nature Reserve Jinggangshan China; 3 Ji ‘an Forestry Bureau, Ji ‘an 343000, China Ji ‘an Forestry Bureau Ji ‘an China; 4 Harvard University Herbaria, 22 Divinity Avenue, Cambridge, MA 02138-2020, USA Harvard University Herbaria Cambridge United States of America

**Keywords:** IUCN, Jinggangshan, Paraphlomideae, phylogenetic

## Abstract

*Paraphlomisjinggangshanensis* (Lamiaceae), a new species from Jiangxi Province, China, is described and illustrated. The new species is morphologically similar to *P.intermedia*, but can be easily distinguished from the latter by its cordate leaf base (*vs.* cuneate, decurrent), stem and calyx tube with glandular hairs (*vs.* short pubescent), and glabrous anthers (*vs.* ciliate anthers). A phylogenetic analysis, based on ITS regions, suggests that *P.jinggangshanensis* represents a separate branch in *Paraphlomis* and is closely related to Clade II. It is currently known only from Jinggangshan National Natural Reserve. Because of its limited distribution and small population size, the species was assessed as Near Threatened (NT) according to the IUCN Red List Categories and Criteria.

## ﻿Introduction

*Paraphlomis* (Prain) Prain, a member of the tribe Paraphlomideae Bendiksby (Lamiaceae: Lamioideae) (Bendiksby et al. 2011; Li et al. 2016; Zhao et al. 2021), is characterized by its herbaceous habit, actinomorphic calyx with five lobes less than half as long as the tube, corolla 2-lipped (1/3) with hairy upper lip but hardly bearded along the margin, included stamens and an apically truncate ovary (Wu and Li 1977; Bendiksby et al. 2011; Ko et al. 2014; Chen et al. 2021). *Paraphlomis* is endemic to eastern and southeastern Asia, including China, India, Indonesia, Korea, Laos, Myanmar, Thailand, and Vietnam (Li and Hedge 1994; Ko et al. 2014; Zhang et al. 2020; Chen et al. 2021).

China, with 23 species documented in the *Flora of China* (Li and Hedge 1994), is the distribution center of *Paraphlomis*. Recently, a number of new species and infraspecies of *Paraphlomis* were described in China, including P.javanicavar.pteropoda D. Fang & K.J. Yan and P.javanicavar.angustifoliaf.albinervia D. Fang & K.J. Yan (Yan and Fang 2009); *P.breviflora* B.Y. Ding, Y.L. Xu & Z.H. Chen (Ding et al. 2019); *P.kuankuoshuiensis* R.B. Zhang, D. Tan & C.B. Ma (Zhang et al. 2020); *P.jiangyongensis* X.L. Yu & A. Liu and *P.coronata* (Vaniot) Y.P. Chen & C.L. Xiang (Chen et al. 2021); *P.nana* Y.P. Chen, C. Xiong & C.L. Xiang (Chen et al. 2022a); *P.longicalyx* Y.P. Chen & C.L. Xiang (Chen et al. 2022b).

During a botanical expedition to Jinggangshan National Nature Reserve, western Jiangxi Province in June 2013, David Boufford and Wen-Bo Liao discovered an unknown species of *Paraphlomis* in Xiangzhou village. Its stem and leaves were densely covered with glandular trichomes and the base of leaves was clearly cordate. Based on its morphological characteristics, which differed from other species of *Paraphlomis*, we suspected that it represented an undescribed species. After carefully comparing it with congeneric specimens, consulting the literature, observing its morphology over two years of additional field investigations (in 2020 and 2021), as well as conducting molecular studies, we confirmed that the species is new to science and formally describe it below.

## ﻿Materials and methods

### ﻿Morphological study

The flowering and fruiting plants of the putative new species were examined in the field and compared with herbarium specimens deposited in A, GH and SYS (herbarium acronyms as in Thiers 2022). All morphological characteristics were measured using dissecting microscopes. Morphological characteristics of similar species of *Paraphlomis* were further observed in digital images of specimens available online at A, GH, KUN, NAS, PE and SYS. Five main characters (habit, leaf shape, calyx, anthers and trichomes) of the putative new species and its most similar species, *Paraphlomisintermedia*, were thoroughly compared.

### ﻿Phylogenetic analyses

The nuclear DNA Internal Transcribed Spacers (ITS) was used for reconstructing the phylogeny of the suspected new species and related taxa based on previous study (Chen et al. 2021; Chen et al. 2022a). Most sequences were downloaded from GenBank, except for the new species, which was newly sequenced in the present study. Genomic DNA of the suspected new species was extracted from silica-gel-dried leaves using the modified 2 × CTAB procedure of Doyle and Doyle (1987). The ITS sequences were amplified with primer pairs ITS4/ITSA, with PCR amplification and sequencing following Chen et al. (2016). A total of 18 accessions, representing 17 species of *Paraphlomis* and one species (*Phlomoidesbracteosa* (Royle ex Benth.) Kamelin & Makhm.) of a related genus were sampled in the phylogenetic study. *Phlomoidesbracteosa* was selected as an outgroup. The GenBank accession numbers are listed in Table 1. Nucleotide sequences were aligned and cleaned using MAFFT 7 (Katoh and Standley 2013). The phylogenetic relationships were assessed using the Maximum Likelihood (ML) method, which was constructed using the program IQ-TREE (Nguyen et al. 2015) with the best-fitting models (TIM+F+G4) chosen according to Bayesian Information Criterion (BIC).

**Table 1.** GenBank accession numbers of the sampled species used in this study.

Species	Voucher	ITS
*Paraphlomisalbida*	A. Liu et al. LK0841 (CSFI); Ningyuan, Hunan, China	MW602124
*Paraphlomisbrevifolia*	L. Wu & W.B. Xu 10965 (IBK); Yangshuo, Guangxi, China	MW602142
*Paraphlomiscoronata*	C.L. Xiang 358 (KUN); Jiangkou, Guizhou, China	MW602123
*Paraphlomisformosana*	Zhong 3676 (E); Taiwan, China	JN680356
*Paraphlomisgracilis*	A. Liu LK0931 (CSFI); Changsha, Hunan, China	MW602134
*Paraphlomishirsutissima*	Fang091060 (KUN); Yunnan, China	EU827096
*Paraphlomishispida*	X. Li LX200702 (GXF); Napo, Guangxi, China	MW602132
*Paraphlomisintermedia*	X. Zhong et al. ZX16823 (CSH); Suichang, Zhejiang, China	MW602135
Paraphlomisjavanicavar.pteropoda	X. Li 2020090501 (GXF); Jingxi, Guangxi, China	MW602140
*Paraphlomisjavanica*	L.B. Jia et al. JLB0029 (KUN); Maguan, Yunnan, China	MW602143
*Paraphlomisjiangyongensis*	A. Liu et al. LK1104 (CSFI); Jiangyong, Hunan, China	MW602129
*Paraphlomisjinggangshanensis*	W.Y. Zhao, Z.C. Liu, Z. Zhang, X.J. Li, ZWY-2060(SYS); Jinggangshan, Jiangxi, China	ON960152
*Paraphlomiskwangtungensis*	Y.P. Chen & Y. Zhao EM1391 (KUN); Huaiji, Guangdong, China	MW602126
*Paraphlomislanceolata*	C.Z. Huang s.n. (KUN); Guidong, Hunan, China	MW602145
*Paraphlomislancidentata*	X. Zhong et al. ZX16824 (CSH); Suichang, Zhejiang, China	MW602136
*Paraphlomismembranacea*	Fang091057 (KUN); Yunnan, China	EU827094
*Paraphlomispaucisetosa*	X.X. Zhu s.n. (KUN); Malipo, Yunnan, China	MW602125
*Paraphlomispaucisetosa*	X. Li LX200704 (GXF); Napo, Guangxi, China	MW602133
*Paraphlomisseticalyx*	A. Liu et al. LK1088 (CSFI); Daoxian, Hunan, China	MW602127
*Phlomoidesbracteosa*	Anders 11464 (M); Afghanistan, Kunar, Chapadarrah	JN680373

## ﻿Results

### ﻿Morphological comparison

In morphology, the putative new species was most similar to *Paraphlomisintermedia* C.Y. Wu & H.W. Li. A comparison of their morphological features is presented in Table 2. These two species share such features as rhizomes with dense fibrous roots, calyx tube obconical, calyx teeth broadly triangular to broadly ovoid triangular and corolla white. The new species, however, differs from *P.intermedia* by its cordate leaf base (*vs.* cuneate, decurrent), stem and calyx tube with glandular trichomes (*vs.* short pubescence), anthers glabrous (*vs.* ciliate). Furthermore, the rhizome of *P.intermedia* has internodes about 1.5–4 cm long (observed in the type specimen), while the rhizome of the putative new species is rather shorter.

**Table 2.** Morphological comparison of *Paraphlomisjinggangshanensis* and *Paraphlomisintermedia*.

Characters	*Paraphlomisjinggangshanensis*	*Paraphlomisintermedia*
**Habit**	erect, stem solitary, unbranched	erect, stem with branches in upper part
**Rhizome**	transverse, internodes 1.5–4 cm	inconspicuous, not transverse
**Trichomes on stem**	puberulent, trichomes retrorse	glandular trichomes erect
**Leaf base**	Cordate	broadly cuneate, abruptly decurrent
**Calyx**	obconical, sparsely pubescent outside	tubular or obconical, with dense glandular trichomes outside
**Anthers**	ovoid, ciliate	ovoid, glabrous
**Nutlets**	sparsely pubescent	glabrous

### ﻿Phylogenetic placement of the putative new species

**Figure 1.**
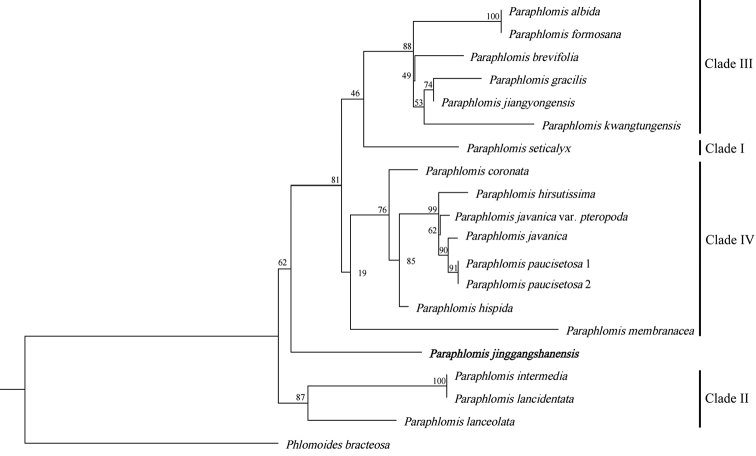
Phylogenetic relationships among 17 species of *Paraphlomis* and *Phlomoidesbracteosa* based on ITS sequences. Numbers above branches indicate Maximum Likelihood bootstraps (ML). The new species described in this study is shown in bold.

The aligned sequences of ITS were 627 bp in length. The resulting phylogenetic tree of *Paraphlomis* in this study was similar to that in a previous study (Chen et al. 2021). Our putative new species formed a separate branch (Fig. 1: ML = 62) that was sister to the previously suggested clades II, and IV by Chen et al. (2021). Fruit morphology is the main factor to distinguish subordinate grades of *Paraphlomis*. Specifically, species of Clade II have glabrous nutlets included in the fruiting calyces, species of Clade III have hairy nutlets, and species of Clade IV share glabrous nutlets that are obviously inflated and exserted from the calyx (Chen et al. 2021). The putative new species was closest to Clade II since its glabrous nutlets were included within the fruiting calyx. However, the putative new species was easily distinguishable from other species in Clade II by being densely covered with glandular trichomes and by the cordate leaf base.

### ﻿Taxonomic treatment

#### ﻿ Paraphlomis jinggangshanensis

Taxon classificationPlantaeLamialesLamiaceae

Boufford, W.B. Liao & W.Y. Zhao sp. nov.

DC980244-BC8D-5CC6-A723-65BA6760AF23

urn:lsid:ipni.org:names:77302738-1

Fig. 2 井冈山假糙苏

##### Type.

China. Jiangxi Province, Jinggangshan City, Jinggangshan National Natural Reserve, roadsides, 26°38'N, 114°15'E, 740 m alt., 10 September 2021, *Wan-Yi Zhao, Zhong-Cheng Liu,Zhong Zhang, XU-Jie Li*, *ZWY-2060* (holotype: SYS!; isotypes: A!, SYS!)

##### Diagnosis.

*Paraphlomisjinggangshanensis* is morphologically similar to *P.intermedia*, but differs by its pubescence of glandular trichomes, cordate leaf base, many-branched stems and glabrous anthers.

##### Description.

**Figure 2.**
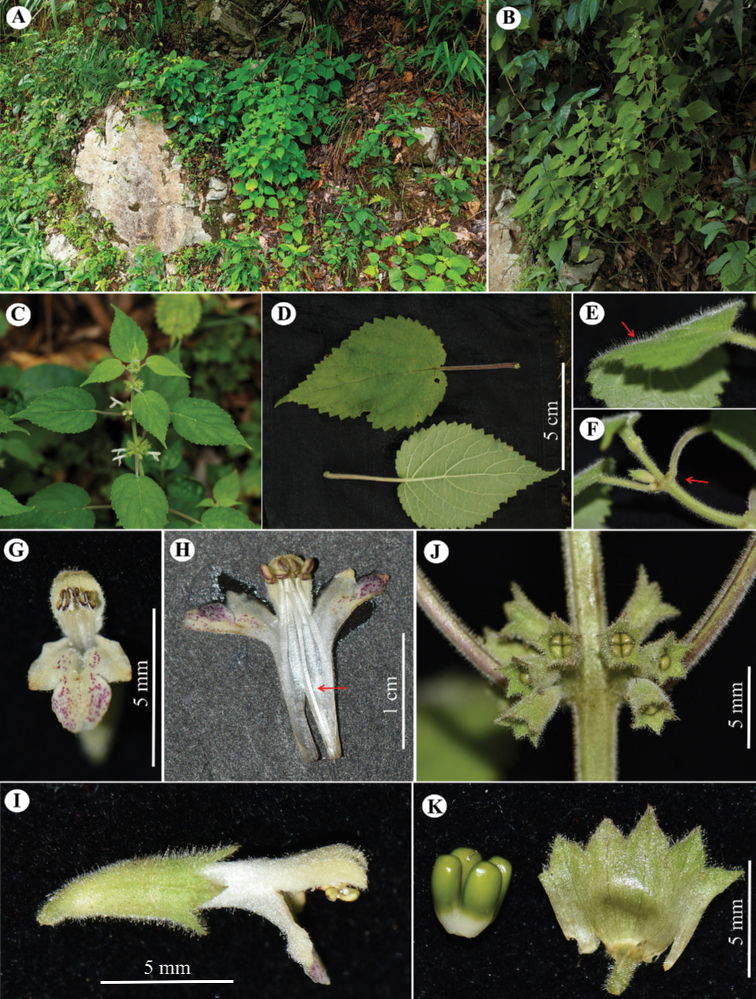
*Paraphlomisjinggangshanensis***A** habit, growing on gravelly hillside **B** plant, stems much branched **C** flowering branch **D** leaves with long petiole, base cordate **E** both surfaces of leaf blade with dense glandular trichomes **F** stem, petiole, and calyx tube with dense glandular trichomes **G** front view of corolla, lower lip dotted with purplish red spots, throat villous annulate **H** inner view of corolla, filaments borne in middle of corolla tube; red arrow indicates glabrous style; anthers glabrous **I** lateral view of flower **J** inflorescence **K** fresh nutlets (glabrous) and inner view of calyx tube (**A–D** by Zhong Zhang **E–K** by Wan-Yi Zhao).

**Herbs**, perennial, 0.4–1.0 m tall. **Rhizomes** short (not transverse), taproot obscure; roots fibrous. **Stems** erect, simple or much branched above middle, 4-angled, grooved, densely covered with short glandular trichomes. **Leaves** opposite; petiole to 9 cm long, with dense short glandular trichomes, green or purplish green; lamina ovate to ovate-oblong, papery, 4–10.2 × 2.5–6.5 cm, base cordate, margin crenate, apex acuminate; abaxially light green, covered with glandular trichomes (more densely so on veins), with glandular spots; adaxially green, densely covered with glandular trichomes, with glandular spots; lateral veins 4 or 5 pairs. **Verticillasters** 10–12 flowered, globose, 2.5–3.0 cm in diam; bracteoles few, ovate-triangular, apex obtuse, ca. 1 mm long, with short glandular trichomes, deciduous; pedicels 1.0–1.5 mm long, or obsolete. **Calyx** green, tubular-obconical, slight curving, ca. 7 mm long, with dense glandular trichomes outside, glabrous except for glandular trichomes on teeth inside, conspicuously 5-veined; teeth 5, subequal, triangular, ca. 1 mm long, apex acute. **Corolla** white, 1.2–1.6 cm long, with dense glandular trichomes outside, pilose annulate in throat inside; tube 8–10 mm long, straight, slightly dilated toward throat, obvious longer than calyx tube; corolla 2-lipped, upper lip oblong, margin entire, ca. 4 mm long, ca. 2.5 mm wide; lower lip 3-lobed, 4–5 mm long, dotted with red spots inside, middle lobe ovate to suborbicular, apex obtuse or retuse, lateral lobes obliquely oblong, apex obtuse. **Stamens** 4, inserted above middle of corolla tube, straight, included, filaments flat, sparsely puberulent-villous; anther cells 2, divergent, ovoid, glabrous. **Style** filiform, included, glabrous, apex subequally 2-lobed. **Ovary** 4-loculed, glabrous. **Nutlets** 4, triquetrous-obovoid, brown at maturity, ca. 2.2 mm long, apex rounded, glabrous. (Fig. 2)

##### Distribution and habitat.

Based on our field observations, *Paraphlomisjinggangshanensis* is located only in Xiangzhou, in the Jinggangshan National Natural Reserve, Jiangxi Province. This area has been considered to be in the subtropical monsoon climate region. *Paraphlomisjinggangshanensis* often occurs in evergreen broadleaved forests along roads above valleys.

##### Conservation status.

This species is currently known to occur only in the Jinggangshan National Natural Reserve in three populations numbering more than two thousand individuals. A road divides the distribution range of *P.jinggangshanensis*. Human activity (such as roadside weed removal) and exotic species have a negative effect on population regeneration. *Paraphlomisjinggangshanensis* is here suggested to be Near Threatened (NT) according to IUCN categories guidelines 10.1 (IUCN Standards and Petitions Subcommittee 2022).

##### Phenology.

Flowering was observed from May to October, and fruiting from July to November.

##### Etymology.

The specific epithet “jinggangshanensis” is derived from the type locality, Jinggangshan National Natural Reserve, Jiangxi Province, China.

##### Additional specimens examined (paratypes).

China. Jiangxi: Jinggangshan City, Jinggangshan National Natural Nature Reserve; NE of the town of Ciping; vicinity of Xiangzhou, roadside, above valley, 26°37'49"N, 114°15'49"E, 545–575 m, 6 June 2013, *David E. Boufford*, *Wen-Bo Liao*, *Bao-Huan Wu*, *Hui-Min Xu & Tian-Tian Yuan 43074* (A); Jinggangshan National Natural Reserve, roadsides, 26°38'N, 114°15'E, 740 m alt., 18 June 2021, *Zhong Zhang Luofu-01* (A, SYS); *ibid*., 15 July 2021, *Zhong Zhang Luofu-06* (A, SYS).

## Supplementary Material

XML Treatment for Paraphlomis jinggangshanensis

## References

[B1] Bendiksby M Thorbek L Scheen AC Lindqvist C Ryding O (2011) An updated phylogeny and classification of Lamiaceae subfamily Lamioideae. Taxon 60(2): 471–484. 10.1002/tax.602015

[B2] Chen YP Drew BT Li B Soltis DE Soltis PS Xiang CL (2016) Resolving the phylogenetic position of *Ombrocharis* (Lamiaceae), with reference to the molecular phylogeny of tribe Elsholtzieae. Taxon 65(1): 123–136. 10.12705/651.8

[B3] Chen YP Liu A Yu XL Xiang CL (2021) A preliminary phylogenetic study of *Paraphlomis* (Lamiaceae) based on molecular and morphological evidence. Plant Diversity 43(3): 206–215. 10.1016/j.pld.2021.03.002 34195505 PMC8233522

[B4] Chen YP Xiong C Zhou HL Chen F Xiang CL (2022a) *Paraphlomisnana* (Lamiaceae), a new species from Chongqing, China. Turkish Journal of Botany 46(2): 176–182. 10.55730/1300-008X.2680

[B5] Chen YP Sun ZP Xiao JF Yan KJ Xiang CL (2022b) *Paraphlomislongicalyx* (Lamiaceae), a new species from the Limestone Area of Guangxi and Guizhou Provinces, Southern China. Systematic Botany 47(1): 251–258. 10.1600/036364422X16442668423572

[B6] Ding BY Chen ZH Xu YL Jin XF Wu DF Chen JB Wu WJ (2019) New species and combination of Lamiaceae from Zhejiang, China. Guangxi Zhi Wu 39(1): 10–15.

[B7] Doyle JJ Doyle JL (1987) A rapid DNA isolation procedure for small quantities of fresh leaf tissue. Phytochemical Bulletin 19: 11–15.

[B8] IUCN Standards and Petitions Subcommittee (2022) Guidelines for Using the IUCN Red List Categories and Criteria. Version 15. Prepared by the Standards and Petitions Subcommittee. https://www.iucnredlist.org/resources/redlistguidelines

[B9] Katoh K Standley DM (2013) MAFFT Multiple Sequence Alignment Software Version 7: Improvements in Performance and Usability. Molecular Biology and Evolution 30(4): 772–780. 10.1093/molbev/mst010 23329690 PMC3603318

[B10] Ko SC Lee YM Chung KS Son DC Nam BM Chung GY (2014) A new species of *Paraphlomis* (Lamiaceae) from Korea: An additional genus to the Korean flora. Phytotaxa 175(1): 51–54. 10.11646/phytotaxa.175.1.6

[B11] Li XW Hedge IC (1994) Lamiaceae. In: Wu ZY Raven PH (Eds) Flora of China, Vol.17. Science Press, Beijing & Missouri Botanical Garden Press, St. Louis, 50–299. http://www.iplant.cn/info/Lamiaceae?t=foc

[B12] Li B Cantino PD Olmstead RG Bramley GLC Xiang CL Ma ZH Tan YH Zhang DX (2016) A large-scale chloroplast phylogeny of the Lamiaceae sheds new light on its subfamilial classification. Scientific Reports 6(1): e34343. 10.1038/srep34343 PMC5066227 27748362

[B13] Nguyen LT Schmidt HA von Haeseler A Minh BQ (2015) IQ-TREE: A fast and effective stochastic algorithm for estimating maximum-likelihood phylogenies. Molecular Biology and Evolution 32(1): 268–274. 10.1093/molbev/msu300 25371430 PMC4271533

[B14] Thiers BM (2022) [continuously updated] Index Herbariorum: A global directory of public herbaria and associated staff. New York Botanical Garden’s Virtual Herbarium. http://sweetgum.nybg.org/science/ih/ [accessed 08.06.2022]

[B15] Wu CY Li HW (1977) *Paraphlomis* Prain. In: Wu CY Li HW (Eds) Flora Reipublicae Popularis Sinicae, vol.65(2). Science Press, Beijing, 545–572.

[B16] Yan KJ Fang D (2009) A supplement to the *Paraphlomis* (Lamiaceae) from Guangxi, China. Redai Yaredai Zhiwu Xuebao 17(7): 91–92. http://jtsb.ijournals.cn/jtsb_cn/article/issue/2009_17_1

[B17] Zhang RB Deng T Dou QL Wei RX He L Ma CB Zhao S Hu S (2020) *Paraphlomiskuankuoshuiensis* (Lamiaceae), a new species from the limestone areas of northern Guizhou, China. PhytoKeys 139: 13–20. 10.3897/phytokeys.139.47055 31997894 PMC6976690

[B18] Zhao F Chen P Salmaki Y Drew BT Wilson TC Scheen AC Celep F Bräuchler C Bendiksby M Wang Q Min DZ Peng H Olmstead RG Li B Xiang CL (2021) An updated tribal classification of Lamiaceae based on plastome phylogenomics. BMC Biology 19(1): e2. 10.1186/s12915-020-00931-z PMC7796571 33419433

